# Dementia-Related Anxiety and General Illness Anxiety Differ Based on Familial Exposure to Persons With Dementia

**DOI:** 10.1093/geroni/igaa057.907

**Published:** 2020-12-16

**Authors:** Erika Fenstermacher, Alexandria Ebert, Natalie Shook, Jerin Lee, Jenna Wilson, Ilana Haliwa

**Affiliations:** 1 West Virginia University, Pursglove, West Virginia, United States; 2 West Virginia University, Morgantown, West Virginia, United States; 3 University of Connecticut, Storrs, Connecticut, United States

## Abstract

Dementia-related anxiety is a specific form of illness anxiety that has been associated with familial exposure to persons with dementia (FMwDs). However, it is unknown whether FMwDs is specifically associated with dementia-related anxiety or whether it is also related to general illness anxiety, which has broader health implications. Furthermore, the level of exposure to family members with dementia may matter. Thus, we examined whether level of familial exposure to dementia was related to general illness anxiety and dementia-related anxiety. Participants (N = 401) aged 18-76 years (M = 39) recruited through Amazon’s Mechanical Turk completed an online survey. Dementia exposure was split into three levels: (1) not knowing a friend/family member with dementia (55.2%); (2) knowing a family member with dementia (33.9%); and (3) providing care for a family member with dementia (10.9%). Familial exposure to dementia was related to both general illness anxiety and dementia-related anxiety. Participants who provided care for FMwDs had significantly higher levels of illness anxiety than both people who had a FMwD and people who did not (ps < .001). Similarly, participants who provided care for FMwDs had significantly higher levels of dementia-related anxiety than participants who did not have a FMwD (p < .01). Caregivers play a critical role in the quality of life of those with dementia, however it is clear that the potential psychological impact of such work is pervasive. This study provides a foundation to explore differences between illness anxiety and dementia worry, and examine interventions to reduce anxiety among caregivers.

